# Service Design Strategies to Enhance Exercise Adherence in Extended Reality Interventions for Older Adults: Systematic Review

**DOI:** 10.2196/86595

**Published:** 2026-04-01

**Authors:** Jiangpan Niu, Yuanyuan Yin, Shan Wang

**Affiliations:** 1 Department of Design Winchester School of Art University of Southampton Winchester United Kingdom

**Keywords:** extended reality, older adults, exercise adherence, service design, systematic review

## Abstract

**Background:**

Exercise has a positive impact on the health of older adults. However, due to physical conditions, psychological factors, and external environment constraints, older adults still face significant challenges in maintaining exercise. Exercise adherence is relatively low. Extended reality (XR) technology offers new ways for older adults to exercise and improve their adherence. Existing research mainly focuses on short-term effects, paying insufficient attention to maintaining long-term engagement and establishing effective incentive mechanisms. By introducing service design methods, user experience, stakeholder collaboration, and adherence support can be better integrated at different stages of exercise intervention, thereby enhancing the willingness and enthusiasm of older adults to continue to participate in exercise.

**Objective:**

This review aims to evaluate how XR‑based exercise interventions targeting populations with a mean age of older than 50 years integrate service design strategies to enhance exercise adherence and to identify critical gaps in their long‑term application.

**Methods:**

A systematic review was conducted across PubMed, Scopus, Web of Science, CINAHL, PsycINFO, and ACM Digital Library (January 2020-July 2025). Eligible studies (1) used XR to support exercise or rehabilitation, (2) included participants with a mean age of older than 50 years, (3) reported at least one service design activity, and (4) provided adherence‑related outcomes. Dual independent screening and structured data extraction were performed.

**Results:**

A total of 9 studies (242 participants) met the inclusion criteria. Most applied participatory, co‑design, or user-centered design with iterative prototyping, but few advanced to full implementation or applied a complete service design cycle. The user experience was generally positive, but evaluations were primarily based on self-reports with limited objective tracking. Although exercise adherence was generally high in most studies (80%-100%), this assessment was primarily based on attendance-related indicators. There was a lack of consistency in how adherence was defined across studies, and no unified measure of exercise adherence was established. Exercise frequency, duration, and attendance were commonly reported, whereas exercise intensity and accuracy were often overlooked. Existing evaluations also lacked long-term tracking of exercise adherence. Regarding intervention delivery settings, most studies were conducted in laboratories, hospitals, and care facilities. Few studies investigated implementation in community settings, which made XR interventions difficult to adapt to the real-world conditions faced by older adults in their daily lives and hindered their promotion and application to a broader community population.

**Conclusions:**

Current XR exercise interventions for older adults show promising short‑term adherence but rarely embed service design continuity or comprehensive adherence monitoring. The combination of structured service design processes with standardized and multidimensional adherence indicators can provide strong support for participants to continue engaging in XR exercise projects. When implemented in community settings, these interventions can enhance scalability and better support an age-friendly XR exercise system.

## Introduction

As life expectancy increases, the older population has grown rapidly, and chronic disease prevalence has risen. Many older adults live with long-term conditions such as cardiovascular disease [1], type 2 diabetes [2], obesity [3], arthritis [4], and dementia [5]. The growing number of such cases is already putting pressure on health care systems globally [6,7]. For example, health care costs for frail older adults range from US $2540 to US $221,400 per person annually [8]. In Singapore, the societal cost associated with multimorbid older adults amounts to US $11,970 per person per year [9]. In Europe and North America, health care expenditures for older adults with multimorbidity are likewise considerable. For example, among older patients with heart failure and diabetes, annual hospital care spending was estimated at approximately US $10,956 per person in England and US $30,877 per person in the United States [10]. Regular exercise helps older adults maintain their health and reduces a variety of age-related problems, such as cardiovascular disease [11,12], decreased musculoskeletal strength [13], risk of falls [14], and psychological distress [15]. Despite the clear benefits of exercise, older adults generally lack the ability to continue exercising [16]. For example, the adherence rate in an aerobic exercise group of older adults averaged only 49.7% [17], while a 6‑month intervention reported a 35% dropout rate among participants aged 65 to 74 years [18].

Older adults face numerous barriers to exercise, including physical fatigue and pain [19,20], psychological concerns and low confidence [21,22], and unsafe or unsuitable spaces in their communities [16,23]. Lack of sustained support and encouragement also makes it difficult for many older adults to maintain long-term exercise [19]. These issues suggest that more flexible interventions that can adapt to individual needs are needed to help older adults cope with physical, psychological, and environmental challenges.

To address these challenges, technologies such as virtual reality (VR), augmented reality (AR), and mixed reality (MR), collectively referred to as extended reality (XR) technology, have been proposed as a potential means of increasing the frequency and accessibility of physical activity among older adults. XR interventions also contain key features such as immersive environments, real-time interactive feedback, and gamification [24-26]. Some studies have reported that VR helps older adults better control blood sugar, improve motor function, and enhance emotional health [25,27,28]. In addition, studies have shown that group activities based on VR decrease loneliness among older adults and increase their enthusiasm and initiative for exercise [29,30]. AR and MR applications support mobility and balance training and are particularly suitable for older adults in institutional care [31,32]. Even though these results are encouraging, most XR-based exercise programs are short-term. Their main goal is to test the usability of the technology, rather than exploring how to sustain long-term behavioral changes [33,34]. Current XR device designs often overlook the needs of older users, leading many to experience difficulties and frustration. Interfaces may be too complex, visuals too intense, or the space required may exceed what their home can provide [35-38].

Since most XR interventions lack long-term planning, service design has gained attention as a way to build more sustainable solutions. It provides a flexible framework to connect user needs, coordinate stakeholders, and maintain consistent experiences over time [39,40]. User journey mapping [41,42], service blueprints [43], and co-creation methods [44] offer ways to identify behavioral touchpoints, track drop-off moments, and target interventions towards a dynamic user experience (UX). However, in many XR exercise studies, service design approaches are often applied in the early design or prototyping stages and rarely receive ongoing support during implementation and evaluation [45,46]. As a result, UXs become fragmented, and behavioral insights are not fully leveraged across the intervention process.

This review responds to existing gaps by taking a service design perspective to explore XR-based physical activity programs for older adults. Adherence is not treated as a fixed individual outcome, but as a dynamic process influenced by ongoing design efforts, emotional involvement, and multisystem collaboration [40,47]. This review adopts the TiSDD (This is Service Design Doing) framework [40] to analyze how service design methods contribute to each phase of XR interventions. The framework includes a 4-stage process covering research, ideation, prototyping, and implementation. Drawing on insights from participatory methods, user-centered design, and health service research, this review examines how sustained adherence is supported and identifies challenges arising from fragmented services. It further informs the development of XR interventions that are attuned to both the needs of older adults and the full exercise engagement cycle.

This review focuses on three core questions:

1. What kinds of service design strategies have been used in XR-based exercise programs for older adults?
2. In what ways are these tools applied to support continuity and improve the user experience throughout the intervention process?
3. What types of adherence outcomes have been observed in these interventions?

## Methods

### Study Design

This study conducted a systematic review to synthesize diverse research, focusing on how XR-based exercise interventions for older adults can address exercise adherence through service design strategies. This review followed the transparent, structured process of the PRISMA (Preferred Reporting Items for Systematic Reviews and Meta-Analyses) guidelines (Multimedia Appendix 1) [48] to ensure reproducibility and methodological rigor.

### Eligibility Criteria

Studies were included if they met all of the following criteria: (1) they enrolled participants with a mean age of older than 50 years—this standard is based on the World Health Organization’s definition of older people in resource-limited settings, where life expectancy is generally shorter [49]; (2) they focused on XR-based interventions involving physical activity, exercise, or rehabilitation; (3) they reported the use of at least one service design method (eg, personas, cocreation, user journey mapping, user-centered design, or participatory design) during the development or implementation of the intervention; (4) they reported outcome data related to exercise adherence, including at least one of the following dimensions: frequency, duration, intensity, or accuracy of exercise performance; and (5) they used or proposed quantitative, qualitative, or mixed method evaluation strategies, with evidence of feasibility, usability, or preliminary outcomes.

Studies were excluded if they met any of the following conditions: (1) they focused solely on cognitive training or entertainment without an exercise component; (2) they did not include older adults as the primary target population; or (3) they were editorials, commentaries, opinion pieces, or lacked sufficient methodological detail or relevance to the research question.

### Information Sources and Search Strategy

A structured search was conducted across 6 academic databases: PubMed, Scopus, Web of Science, CINAHL, PsycINFO, and the ACM Digital Library. The search strategy combined Boolean operators and thematic clusters covering: XR technologies (“virtual reality” OR “augmented reality” OR “mixed reality” OR “extended reality” OR “immersive technology”), service design methods (“service design” OR “user journey” OR “journey mapping” OR “experience map” OR “co-design” OR “participatory design” OR “user-centered design” OR “human-centered design”), and the target population and activity (“older adult” OR “elderly” OR “geriatric” AND “exercise” OR “physical activity” OR “rehabilitation”). Searches were limited to peer-reviewed papers published in English between January 2020 and July 2025. Full-text availability was required for eligibility assessment and data extraction; records for which the full text could not be accessed were excluded at the screening stage. Gray literature sources and trial registries were not systematically searched, as this review focused on peer-reviewed studies that provided sufficient methodological detail on service design activities and XR intervention implementation. The full database-specific search strategies are detailed in Multimedia Appendix 2.

### Study Selection

All retrieved records were imported into a Zotero library (version 7.0.24; Corporation for Digital Scholarship), and duplicates were removed. Two independent reviewers (JN and SW) screened the titles, abstracts, and full texts of the remaining papers against the predefined inclusion and exclusion criteria. Discrepancies were resolved through discussion; if consensus could not be reached, a third reviewer (YY) was consulted.

### Data Extraction and Management

Data extraction was performed using a standardized Microsoft Excel 2021 spreadsheet to ensure consistency across studies. Two reviewers independently extracted data on study characteristics, service design strategies, UX, and adherence outcomes. Discrepancies were resolved through discussion until consensus was reached. To classify the application stages of service design strategies, this study adopted the TiSDD framework [40]. Table 1 summarizes the definitions and typical activities of each stage, which served as the reference for coding service design applications in the included studies.

**Table 1:** Service Design Stages Defined by the TiSDD (This is Service Design Doing) framework.

Stage	Description	Typical activities
Research	Understanding users, behaviors, and context	Ethnography, interviews, observation, participatory workshops, and data analysis
Ideation	Generating, refining, and prioritizing concepts	Brainstorming, clustering, voting, and decision matrices
Prototyping	Developing and testing service concepts	Storyboarding, role-playing, mock-ups, and digital/physical prototypes
Implementation	Deploying and managing interventions	Change management, technical development, training, and monitoring

### Data Synthesis

Data synthesis was organized around 3 research questions and divided into 4 sections, including study characteristics, service design outcomes, UX outcomes, and adherence outcomes. This thematic framework enabled a narrative synthesis that examined how service design tools were used, how they supported continuity and UX, and what types of adherence outcomes were reported.

### Quality Assessment

To assess the quality of the studies, critical appraisal was conducted using the Mixed Methods Appraisal Tool (MMAT; version 2018; McGill University) [50]. The MMAT is a specially designed tool that can be used to assess the quality of different types of studies in the same review, including qualitative, quantitative, and mixed methods studies. The quality of all studies was assessed by the first reviewer (JN) and checked for accuracy by 2 other authors (YY and SW). In line with MMAT guidance, the tool was not used to score or exclude studies, but to support the interpretation of the findings [50].

## Results

### Sample

**Figure 1:**
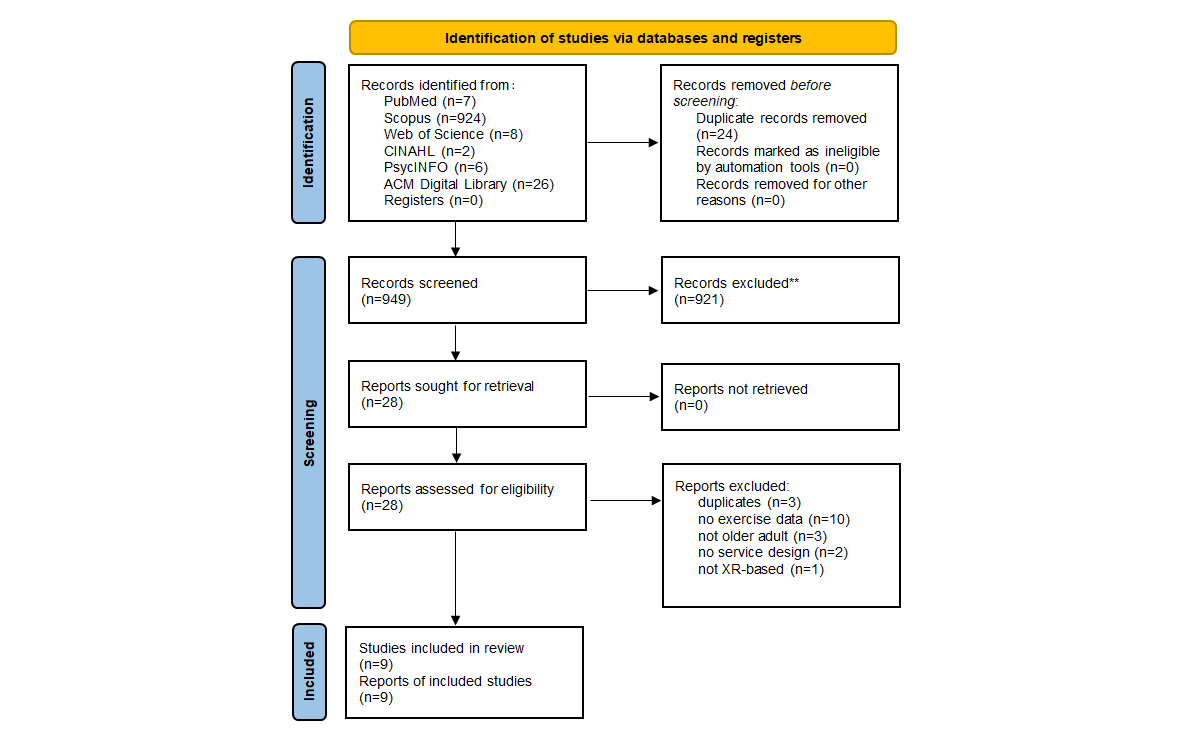
PRISMA (Preferred Reporting Items for Systematic Reviews and Meta-Analyses) 2020 flow diagram of the study selection process.

The total number of identified records was 973. After duplicates were removed, 949 records remained, and these were screened for title and abstract relevance. The number of excluded studies after title and abstract screening was 921. The full texts of the remaining 28 papers were read and analyzed, of which 19 were excluded due to factors such as the absence of exercise-related data, duplication, non–older adult populations, lack of service design elements, or lack of XR-based interventions. As a result, 9 studies were included in the final review (Figure 1).

### Study Characteristics

This review included 9 studies published between 2020 and 2025, conducted across 9 countries: Italy, Portugal, the Netherlands, the United Kingdom, Germany, Spain, Canada, Cyprus, and China. Of the 9 studies, 67% (6/9) were mixed methods designs [51-56], 11% (1/9) was a mixed methods study with an embedded randomized controlled trial [57], 11% (1/9) was a quasi-randomized controlled trial [58], and 11% (1/9) was a nonrandomized quantitative study [59]. A total of 242 participants were involved across all studies, with sample sizes ranging from 6 to 75. The mean age of participants ranged from 52.8 to 86.8 years. A total of 89% (8/9) included both male and female participants [51-54,56-59], while 11% (1/9) did not report gender information [55].

All 100% (9/9) studies used VR as the XR modality [51-59]. A total of 67% (6/9) of studies reported specific XR headset models [51-53,55,56,58], while 33% (3/9) did not specify the hardware used [54,57,59]. A total of 78% (7/9) of studies described the XR interaction modality [51,52,54-58], whereas 22% (2/9) did not report interaction details [53,59]. A total of 100% (9/9) of studies reported some form of XR supervision and support during intervention delivery [51-59]. A total of 33% (3/9) of studies reported information on XR cybersickness or tolerability [51,54,55], while 67% (6/9) did not report tolerability-related data [52,53,56-59].

Exercise types included 44% (4/9) upper limb rehabilitation interventions [51,52,55,56], 22% (2/9) gait or balance training [54,58], 11% (1/9) lower limb training [57], 11% (1/9) adapted physical activity [59], and 11% (1/9) multidomain physical-cognitive rehabilitation [53].

Intervention settings included 44% (4/9) hospital-based studies [51-53,57], 11% (1/9) university or research laboratory [58], 11% (1/9) biomedical research institute [54], 11% (1/9) long-term care residence [56], and 11% (1/9) home-based environment [55], 11% (1/9) study used a mixed-setting intervention across nursing homes, hospitals, and home environments [59] (Table 2).

**Table 2:** Study characteristics.

Author (year), country	Study design	Sample size	Age (years)	Sex	XR^a^ type	XR hardware	XR interaction Modality	XR supervision and support	XR cybersickness / tolerability	Exercise type	Intervention setting
De Luca et al [59], (2025), Italy, Portugal, Netherlands	Nonrandomized quantitative	75	Mean age 79.8 (SD 9.96)	F^b^ and M^c^	VR^d^	Not reported	Not reported	Trainer- or coach-supported use	Not reported	Adapted physical activity	Nursing homes, hospitals, and home settings
Sweeney et al [53], (2025), United Kingdom	Mixed methods	60	Mean age 69.6 (SD 14.0)	F and M	VR	Meta Quest headset	Not reported	Supervised group-based sessions with rehabilitation staff	Not reported	Multitechnology rehabilitation training: upper limb, lower limb, balance, cognitive, and whole-body functional training	Hospital-based intervention
Ciemer et al [58], (2025), Germany	Quasi-RCT^e^	32	Mean age 70.00 (SD 3.33)	F and M	VR	HTC Vive Pro 2	Walking-based interaction	On-site supervision by 2 trained supervisors, with real-time monitoring via an external screen	Not reported	Gait-based fall prevention exergame	University laboratory
Bosch-Barceló et al [54], (2024), Spain	Mixed methods	8	Mean age 61 (SD 9)	F and M	VR	Not reported	Treadmill-based gait training with foot tracking	Supervised by physiotherapists	Fatigue and sweating	Gait training (treadmill walking + dual-task intervention)	Biomedical Research Institute
Mehrabi et al [55], (2022), Canada	Mixed methods	12	≥60	None	VR	Oculus Quest 2	Hand-held controllers	Remote support	Potential challenges related to nausea and motion sickness	upper-body physical activity	Home-based program
Matsangidou et al [51], (2022), Cyprus	Mixed methods	20	Mean age 77.85 (SD 11.35)	F and M	VR	VIVE Pro-Eye VR; Vive Trackers	Touch-based interaction without buttons, where objects in the VR environment are triggered by direct contact	On-site in-person supervision by physiotherapists, with real-time monitoring via an external screen	Motion sickness and anxiety noted as potential issues	Upper-limb training	Hospital setting
Matsangidou et al [52], (2023), Cyprus	Mixed methods	7	Mean age 81.14 (SD 3.84)	F and M	VR	VIVE Pro-Eye VR; Vive Trackers	Hand-held controllers and Vive Trackers with gaze tracking	On-site in-person supervision by physiotherapists, with real-time monitoring via an external screen	Not reported	Upper limb rehabilitation exercises	Hospital setting
Eisapour et al [56], (2020), Canada	Mixed methods	6	Mean age 86.8 (SD 6.2)	F and M	VR	Oculus Rift CV1	Hand-held touch controllers; audio (voice) instructions	Supervised by exercise therapists and researchers	Not reported	Upper limb exercises	Long-term care residence
Xu et al [57], (2021), China	Mixed methods study with an embedded RCT^f^	22	Mean age in the intervention group is 57.55, and in the control group is 52.82	F and M	VR	Not reported	Depth camera-based motion tracking	Supervised by physical therapists	Not reported	Lower limb functional training	Hospital setting

^a^XR: extended reality.

^b^F: female.

^c^M: male.

^d^VR: virtual reality.

^e^Quasi-RCT: quasi–randomized controlled trial.

^f^RCT: randomized controlled trial.

### Quality Assessment of the Studies Included

Based on the MMAT assessment, the overall methodological quality of the included studies was acceptable.

Most studies demonstrated adequate quality across their respective methodological domains. The qualitative components generally met MMAT criteria, showing appropriate study design, data collection, and coherence between data, analysis, and interpretation.

Among quantitative studies, one randomized controlled trial met all MMAT criteria. Several nonrandomized studies showed limitations related to confounding, as confounders were not clearly accounted for in the study design or analysis.

Most mixed methods studies provided a clear rationale for using a mixed methods design and demonstrated integration between qualitative and quantitative components. However, some studies showed limited methodological transparency, reflected by “can’t tell” ratings on selected MMAT criteria.

No studies were excluded on the basis of quality appraisal. Detailed MMAT ratings for each study are provided in Multimedia Appendix 3 [51-59].

### Service Design Outcomes

#### Service Design Tools and Strategies

Across the 9 included studies, service design strategies were predominantly grounded in participatory, co‑design, and user‑centered approaches. A total of 4 (44%) studies applied participatory design [52,56,57,59], 2 (22%) studies adopted co-design [51,53], 1 (11%) study used collaborative design [58], 1 (11%) study adopted user‑centered design [54], and 1 (11%) study explicitly combined participatory and user‑centered methods [55].

Stakeholder involvement demonstrated a multistakeholder pattern in most cases. All studies (9/9, 100%) involved older adults [51-59], 8 (89%) studies engaged rehabilitation professionals such as physical therapists, rehabilitation physicians, or kinesiologists [52-59]. Five (56%) studies involved technical and design experts [51,52,54,55,58], 4 (44%) studies included medical staff such as physicians, nurses, or paramedical professionals [51,53,54,59], and 1 (11%) studies engaged caregivers or community staff [59]. In total, 7 (78%) studies incorporated 3 or more stakeholder groups [51-55,58,59], reflecting a predominant emphasis on collaborative development across clinical, technical, and end user perspectives (Table 3).

**Table 3:** Service design tools and applications across service stages.

Author (year)	Service design strategy	Stakeholder groups involved	Application stage (TiSDD^a^ model: research/ideation/prototyping/implementation)	Service design activities and functions
De Luca et al [59], (2025)	Participatory design	Older adults, caregivers, therapists, communities, and clinicians.	Research + Prototyping: Participatory design was used to engage older adults, caregivers, therapists, and other stakeholders to identify user needs and define goals (Research), iteratively refined the system prototype based on user feedback during development (Prototyping).	Service design activities structured adapted physical activity services for older adults in nursing homes and home settings by defining service workflows and delivery scenarios, positioning the VR^b^ system as part of an integrated service rather than a stand-alone technology.
Sweeney et al [53], (2025)	Cocreation	Older adults with stroke, physicians, nurses, and therapists.	Implementation: The intervention was deployed, managed, and monitored in a real-world hospital ward environment.	Service design activities structured a multitechnology, group-based rehabilitation exercise service within an acute stroke unit by configuring service workflows, defining staff and patient roles, and integrating multiple rehabilitation technologies into a supervised group delivery model.
Ciemer et al [58], (2025)	Collaborative	Older adults, expert groups from the domains of Human Movement Science, Experience Design, and Game Design.	Research + Prototyping: User needs were identified through involvement of target users (Research), and the EXploVR prototype was developed and iteratively tested by a multidisciplinary team together with older adults (Prototyping).	Service design activities structured EXploVR as a supervised, community-oriented fall-prevention service by defining training workflows, staff roles, spatial and safety arrangements, and designing the intervention to support relevance to daily-life mobility and participation for older adults.
Bosch-Barceló et al [54], (2024)	User‑centered design	Older adults with Parkinson disease, physiotherapists, nurses, and computing scientists.	Research + Prototyping + Implementation: Initial user needs were collected through feedback and expert interviews (Research), software was developed and validated using the Scrum method (Prototyping), and implementing the intervention in a real world setting and collecting feedback (Implementation).	Service design activities structured a supervised, technology-supported gait rehabilitation service by defining session workflows, physiotherapists and patient roles, safety arrangements, and progression rules, integrating a gamified VR environment into routine physiotherapy-led delivery.
Mehrabi et al [55], (2022)	Participatory and user-centered design	Older adults with no or mild cognitive impairment, exercise professionals, game designers, engineers from human factors and assistive technology fields, and a local VR studio.	Research + Prototyping: An extensive user-centered and participatory design process was applied, involving stakeholders including older adults, exercise professionals, game designers, and assistive technology experts (Research). The system was iteratively refined based on user feedback (Prototyping). The paper did not detail each phase separately.	Service design activities structured a remotely delivered home-based VR exercise service by defining session workflows, participant and staff roles, and remote onboarding and support procedures, enabling older adults to safely engage in a 6-week exergame intervention outside clinical settings.
Matsangidou et al [51], (2022)	Co-design	Older adults with dementia, medical and paramedical staff, Human-Computer Interaction (HCI) in health care experts.	Research + Ideation + Prototyping: Needs and barriers were identified through focus groups (Research), followed by concept generation and prioritization through workshops and brainstorming sessions (Ideation), and then rapid prototyping and testing with user feedback (Prototyping).	Service design activities structured a supervised VR-based physical training service for people living with dementia by defining session workflows, therapist-patient roles, safety and calibration procedures, and integrating VR into routine care delivery within a restricted hospital environment.
Matsangidou et al [52], (2023)	Participatory design	Older adults with dementia, physiotherapist, human-computer interaction (HCI) researcher.	Prototyping: The intervention applied the RITE rapid prototyping method, using observation notes and semistructured interviews to iteratively refine the system (Prototyping).	Service design activities structured a personalized, therapist-supported VR rehabilitation service for seated upper-body physical training for people living with dementia, by defining calibration-based exercise workflows, therapist monitoring roles, and adaptive feedback mechanisms to support safe and independent task execution in clinical settings.
Eisapour et al [56], (2020)	Participatory design	Older adults with dementia, exercise therapists, and kinesiologists.	Research + Prototyping: User needs were gathered through focus groups and observations (Research), and the prototypes were refined iteratively based on user feedback (Prototyping).	Service design activities structured dementia-friendly VR exercise delivery by co-designing with therapists and people living with dementia, iteratively refining 2 VR exercise environments, and embedding safety and accessibility supports (eg, audio guidance and individualized calibration) for seated upper-body training in long-term care.
Xu et al [57], (2021)	Participatory design	Older adults with stroke, physical therapists, and rehabilitation physicians.	Research + Prototyping + Implementation: Design inputs were collected from stroke patients, therapists, and physicians (Research); the game prototype was refined based on feedback to improve usability (Prototyping); and the final version was tested in a hospital rehabilitation ward with evaluations from multiple stakeholder perspectives (Implementation).	Service design activities structured a therapist-supervised VR service for poststroke lower-limb rehabilitation by translating stepping- and gait-training protocols into adjustable Stomp-Joy–based game tasks, and embedding role-based clinical workflows for session prescription, progression control, and safety supervision.

^a^TiSDD: This is Service Design Doing.

^b^VR: virtual reality.

#### Application Across Service Stages

The coverage of service design stages demonstrated apparent variation (Table 3). Seven of 9 (78%) studies applied the Research stage, identifying user needs and design elements through interviews, focus groups, or on-site observations [51,54-59]. One (11%) study addressed the Ideation stage, using workshops and brainstorming sessions to generate and prioritize concepts [51]. Eight (89%) studies engaged in the Prototyping stage, improving the usability and interaction experience of the system through rapid prototyping or RITE methods [51,52,54-59]. Three (33%) studies entered the implementation stage, deploying and managing the system in hospital, biomedical research, or rehabilitation ward settings [53,54,57].

Regarding stage coverage, 2 (22%) studies were single-stage [52,53] and focused exclusively on Implementation or Prototyping. A total of 7 (78%) studies were multistage, involving 2 or more phases [51,54-59]. Among these, 4 (44%) studies adopted a 2-stage process from the Research to Prototyping stage, enabling user needs to be identified and rapidly translated into iterative prototypes [55,56,58,59]. Two (22%) studies implemented a 3-stage combination of Research, Prototyping, and Implementation [54,57], 1 (11%) followed a 3-stage process covering the sequence of Research, Ideation, and Prototyping [51]. Notably, no study completed all 4 stages of the TiSDD model.

Across the included studies, service design activities were primarily used to configure how XR interventions functioned as services in real-world care contexts. Specifically, service design activities [51-59] were used to (1) define service workflows and delivery scenarios, (2) clarify roles and responsibilities of patients, therapists, and staff, and (3) integrate XR systems into existing rehabilitation, care, or home service structures (Table 3).

### User Experience Outcomes

The included studies evaluated UX using 5 methods. Five of the 9 (56%) of studies used standardized usability questionnaires, such as System Usability Scale, The Single Ease Question, Simulator Sickness Questionnaire, Questionnaire for User Interaction Satisfaction, and NATU Quest (Assistive Technology Usability Questionnaire for people with Neurological diseases), to assess system usability and task difficulty [51,52,54,55,59]. Five of the 9 (56%) studies evaluated emotional and immersive experience through tools like Flow Short Scale, Physical Activity Enjoyment Scale, Exergame Enjoyment Questionnaire, Physical Activity Affect Scale, Visual Analogue Scale, Observed Emotion Rating Scale, and Slater-Usoh-Steed Questionnaire [51,52,55,56,58]. Two of the 9 (22%) studies captured motivation and engagement using the intrinsic motivation inventory and a 4-point engagement scale [56,57]. Two of the 9 (22%) of studies examined self-efficacy and safety perceptions using tools such as ABC-6/8, the Bandura scale, and custom safety items [55,58]. Six of the 9 (67%) studies used qualitative feedback methods, including interviews and open-ended forms [52-57].

Participants generally reported a positive UX. Five of the 9 (56%) studies reported that interventions were enjoyable and immersive, with participants frequently highlighting high levels of engagement and emotional benefits [51,52,54,56,58]. Three of the 9 (33%) of studies indicated perceived usefulness and value for rehabilitation, with self‑report measures reflecting strong motivation and recognition of exercise benefits [55,57,59]. Two of the 9 (22%) studies included feedback from clinical staff who found the interventions both feasible and encouraging [53,57]. They highlighted outcomes such as increased patient confidence, improved mood, and reduced staff workload. However, 33% (3/9) of studies also pointed to negative aspects, including fatigue, discomfort from wearing head-mounted displays, and a decrease in enjoyment during cognitively demanding or extended sessions [51,54,57] (Table 4).

**Table 4:** User experience (UX) outcomes.

Author (year)	UX measurement method	UX feedback
De Luca et al [59], (2025)	QUIS7^a^-based questionnaire (5-point Likert)	76.7% (58/75) reported positive experience, 68.5% (51/75) were satisfied, 65.8% (49/75) found it useful.
Sweeney et al [53], (2025)	Staff interviews (qualitative)	Clinical staff considered the intervention acceptable, reporting that it improved patients’ confidence, mood, and motivation for rehabilitation, while also alleviating staff workload.
Ciemer et al [58], (2025)	FKS^b^, PACES-S^c^, EEQ^d^, ABC-6/8^e^, custom questions on confidence	High levels of enjoyment (FKS, PACES, EEQ scores high and stable); participants reported the training as enjoyable, immersive, meaningful, and safe, with reduced fear of falling.
Bosch-Barceló et al [54], (2024)	SUS^f^, NATU Quest^g^, SSQ^h^, user feedback	High satisfaction reported. participants preferred natural environments (countryside, forest), found the experience pleasant and immersive, and appreciated the dog design as reassuring and the environments as realistic. Some participants noted that fog and high speeds reduced enjoyment. Although they reported fatigue, the system was generally acceptable and motivating.
Mehrabi et al [55], (2022)	Ad hoc usability and game UX questionnaire, 5-point pictorial Likert enjoyment scale, PAAS^i^, Bandura self-efficacy scale, semi-structured interviews	Positive UX: high satisfaction, usability, and immersion; high enjoyment (Likert); interviews show game is engaging and feasible; PAAS indicates positive emotions; self-efficacy supports future exercise intention.
Matsangidou et al [51], (2022)	OERS^j^, VAS^k^, VRSQ^l^, SUS, SEQ^m^	FIVR^n^ significantly increased positive affect (higher OERS scores), reduced sadness and anxiety; patients reported the training to be more enjoyable, immersive, and emotionally positive; SIVR^o^ was better than conventional training but less effective than FIVR; some patients remained sensitive to wearing the HMD^p^.
Matsangidou et al [52], (2023)	SUS, SEQ, OERS, VAS, task performance, and interviews	In the final prototype, significant improvements were observed in presence and usability (with SUS and SEQ scores significantly increased); participants showed increased positive emotions (pleasure) and reduced negative emotions (such as sadness, anxiety, anger); observation notes and interviews indicated that VR training elicited feelings of enjoyment, interest, and self-identification (eg, perceiving the training as more relevant to their personal context and natural to perform).
Eisapour et al [56], (2020)	Daily and weekly questionnaires (5-point Likert), 4-point engagement scale, interviews, open-ended feedback, and expert reviews.	Participants generally reported enjoying the VR experience, finding it relaxing and enjoyable, with high immersion, comfort, and usability; some expressed a preference for more challenging tasks or more variety rather than repeating the same movement.
Xu et al [57], (2021)	IMI^q^ questionnaire (5 dimensions), semistructured interviews	Patients reported high scores in interest/enjoyment, perceived competence, and value/usefulness on the IMI questionnaire, indicating high engagement and perceived benefits of the game. However, it was noted that interest may decline with long-term use and that the system lacked a recording mechanism. Both patients and clinicians considered the experience pleasant and motivating.

^a^QUIS7: Questionnaire for User Interaction Satisfaction.

^b^FKS: Flow Short Scale.

^c^PACES-S: Physical Activity Enjoyment Scale.

^d^EEQ: Exergame Enjoyment Questionnaire.

^e^ABC-6/8: Activities-specific Balance Confidence Scales.

^f^SUS: System Usability Scale.

^g^NATU Quest: Assistive Technology Usability Questionnaire for people with Neurological diseases.

^h^SSQ: Simulator Sickness Questionnaire.

^i^PAAS: Physical Activity Affect Scale.

^j^OERS: Observed Emotion Rating Scale.

^k^VAS: Visual Analogue Scale.

^l^VRSQ: Slater-Usoh-Steed Questionnaire.

^m^SEQ: The Single Ease Question.

^n^FIVR: fully immersive virtual reality.

^o^SIVR: Semi Immersive VR.

^p^HMD: Head-Mounted Display.

^q^IMI: Intrinsic Motivation Inventory.

### Exercise Adherence Outcomes

This review analyzed exercise adherence outcomes reported across the included studies. Given the heterogeneity in adherence definitions and measurement approaches, no conversion or standardization of adherence metrics was performed. Instead, adherence outcomes were synthesized by grouping indicators according to their underlying behavioral constructs, allowing comparison within similar categories while highlighting dimensions that remain underevaluated.

Attendance-based adherence indicators were the most consistently reported. Five of the 9 (56%) studies reported attendance-related indicators, including completion and dropout rates, with completion rates ranging from 80% to 100% and dropout rates varying between 2% and 13% [53,55-58]. In this review, references to “high adherence” were primarily derived from attendance-related indicators, as these were the most frequently and consistently reported measures across studies.

Performance-based adherence indicators were reported less consistently and captured different aspects of task execution and progression. Four of the 9 (44%) of studies monitored exercise accuracy, including task precision, movement quality, and success rates through in-game metrics or trajectory tracking systems [51,52,54,56]. Furthermore, 33% (3/9) of studies discussed performance progression across sessions, including observed improvements or declines in task success rates and response times [52-54].

Self-reported adherence indicators were the least frequently reported. One of the 9 (11%) studies assessed self-reported perceptions of adherence, with 65.8% (49/75) of participants indicating that the system supported their physical activity routines [59].

Across the 4 core adherence components synthesized in this review, including frequency, duration, intensity, and accuracy [60]. Seven of the 9 (78%) of studies clearly defined exercise frequency [51,52,54-58]. Furthermore, 89% (8/9) of studies reported the duration of exercise, including both session length and program length [51-58]. Three of the 9 (33%) studies addressed exercise intensity [55,56,59]. Four of the 9 (44%) of studies captured the accuracy of exercise performance [51,52,54,56]. None of the studies included long-term follow-up to assess sustained exercise adherence.

No studies were excluded from the review due to unclear adherence reporting; however, the variability and incompleteness of adherence measures limited cross-study comparability and constrained conclusions regarding long-term adherence (Table 5).

**Table 5:** Exercise adherence outcomes.

Author (year)	Exercise protocol (planned dose)	Exercise adherence indicators	Exercise adherence results	Follow-up
De Luca et al [59], (2025)	6-week program	IPAQ-SF MET^a^ value (quantitative), IPAQ-SF^b^ activity level classification (inactive/active), self-reported improvement in physical activity adherence.	No significant improvement in overall physical activity levels; 9% (7/75) of participants improved from inactive to sufficiently active; 65.8% (49/75) agreed and 9.6% (7/75) strongly agreed that VR2Care improved adherence to physical activity.	Not reported
Sweeney et al [53], (2025)	Duration not specified	Session attendance, training duration, task completion rate, performance progression.	On average, each patient completed 9.1 sessions (SD 7.4) during hospitalization, with a total rehabilitation time of 508.4 minutes (SD 521.2) and an average of 52.0 minutes per session (SD 15.7). Patients achieved an additional 52 minutes of rehabilitation per day prior to discharge. Dropout rate was low (1/60, 2%).	Not reported
Ciemer et al [58], (2025)	3-week program; 2 sessions per week; 60 minutes per session; total of 6 sessions	Adherence was tracked through an attendance log recorded at each session.	A total of 32 participants completed the study, with 16 in the intervention group and 16 in the control group. A total of 6 participants dropped out during the study (intervention group: n=4; control group: n=2), resulting in an overall dropout rate of only 13% (4/32).	Not reported
Bosch-Barceló et al [54], (2024)	3 sessions; 1 session per week; each session included treadmill warm-up and 4 bouts of gait training with rest intervals; total session duration approximately 20-21 minutes	Obstacle avoidance performance (success rate in each bout across 3 different difficulty environments).	Obstacle avoidance performance improved during the training phases, with better performance after familiarization on Day 1; performance slightly decreased on Days 2 and 3 as difficulty increased, but overall remained satisfactory.	Not reported
Mehrabi et al [55], (2022)	6 weeks; 3 sessions per week; 15-20 minutes per session	Session adherence rate; gameplay metrics.	The authors anticipated an adherence rate of ≥80%. Gameplay performance metrics were automatically recorded to assess participation.	Not reported
Matsangidou et al [51], (2022)	3 experimental sessions; each lasting approximately 30 minutes	Task accuracy (number of correctly performed repetitions out of 10), task independence (number of therapist interventions required), completion time (seconds), movement quality (match with baseline movements).	Under the FIVR^c^ system, patients demonstrated higher task accuracy, greater independence, and better movement quality; the time required for simple movements (Side Arm Raise) was shorter, and although the time for complex movements (Overhead Press) was longer, accuracy improved.	Not reported
Matsangidou et al [52], (2023)	3 iterations of RITE^d^ testing; each iteration approximately 30 minutes, including up to 20 minutes of VR^e^ exposure	Task completion rate, task independence score, execution time, movement trajectory error.	Over the 3 prototype iterations, task completion and independence significantly improved, execution time decreased, and movement trajectories became closer to the target, indicating progressively improved exercise adherence.	Not reported
Eisapour et al [56], (2020)	3-week program; 5 sessions per week; ≈20 minutes per session	Completion rate (whether all exercises were completed), range of motion (reach), movement distance, velocity.	All participants completed all scheduled VR exercise sessions (6/6, 100% completion); exercise performance (range of motion, distance, velocity) was comparable to therapist-led exercise, with no significant differences; participants perceived VR exercise workload as adequate and comparable to therapist-led sessions.	Not reported
Xu et al [57], (2021)	2-week program; 5 sessions per week; VR training (30 minutes) combined with conventional physical therapy; total of 10 sessions	Intervention attendance/dropout rate; number of completed sessions during the intervention period.	Of the 24 participants, 2 withdrew—one due to a fall during training, without injury, and one for personal reasons.	Not reported

^a^IPAQ-SF MET: International Physical Activity Questionnaire Short Form - Metabolic Equivalent of Task.

^b^IPAQ-SF: International Physical Activity Questionnaire Short Form.

^c^FIVR: fully immersive virtual reality.

^d^RITE: Rapid Iterative Testing and Evaluation.

^e^VR: virtual reality.

## Discussion

### Principal Results

This review synthesized evidence from 9 XR‑based exercise interventions targeting older adults and revealed a field that was promising yet constrained by structural and methodological limitations. Most studies grounded their service design strategies in participatory, co‑design, and user‑centered approaches [51-57,59], frequently combined with iterative prototyping [51,54-59]. However, service design processes were often incomplete, with most studies concentrating on early research and prototyping stages, showing limited engagement in ideation [51] and progression to implementation. Only 3 studies progressed to the Implementation stage [53,54,57]. Although multiple stakeholders were involved and participatory principles were widely adopted, their contributions were largely confined to early-stage design decisions and usability refinement, rather than extended to shaping long-term service integration or delivery models. This uneven coverage across service stages constrained the translation of interventions into sustained service offerings. When interpreted through behavior change frameworks such as Capability, Opportunity, Motivation–Behavior [61] and behavior change techniques [62], the limited continuity of service design across later stages helps explain why sustained exercise behavior was rarely examined. While early-stage co-design and prototyping supported initial engagement and motivation, fewer interventions addressed opportunity-related factors, such as integration into everyday contexts, reinforcement strategies, and long-term support mechanisms, which are essential for maintaining behavior over time.

UX outcomes were generally positive. Participants reported pleasant experiences, high levels of immersion, and increased motivation in questionnaires, usability evaluations, and interviews. However, some studies also noted negative adverse effects, such as fatigue, headset discomfort, and decreased enjoyment during challenging or long tasks [51,54,57]. Overall, most evaluations relied on subjective reports, with limited use of objective measures, such as physiological or behavioral data. This reliance on self-reported short-term experience indicators limits the extent to which positive UX can be interpreted as evidence of sustained engagement. While enjoyment and immersion appear effective in supporting initial participation, the lack of longitudinal and objective measures constrains understanding of how these experiences translate into continued exercise behavior over time.

Most studies reported high levels of exercise adherence, though inconsistently across dimensions, despite the use of a 4D adherence framework [60] in our synthesis; few studies comprehensively reported across all dimensions. Frequency and duration were the most reported measures of exercise adherence; however, few studies compared them with established exercise guidelines. Most studies conducted minimal monitoring of exercise intensity, and only 1 applied validated physiological measurement. While some VR systems included metrics related to movement accuracy, the studies did not integrate these metrics into the formal outcome framework for exercise adherence. This fragmentation of adherence dimensions impeded cross-study comparison and masked the contribution of specific design features to behavioral engagement. Most studies assessed exercise adherence using non‑standardized, short‑term measures. Attendance and task completion were the most commonly used surrogate measures of adherence; some studies also reported adherence through in-game performance metrics, performance progression over time, or self-reported perceptions of adherence, but none adopted a standardized long-term assessment framework. As a result, the consistently high adherence rates reported across studies should be interpreted cautiously, as they primarily reflect short-term participation rather than sustained exercise continuity. The absence of standardized, multidimensional, and longitudinal adherence assessments limits insight into whether engagement persisted beyond the intervention period.

Notably, 8 studies conducted interventions in hospital, laboratory, or long‑term care settings [51-54,56-59], and 1 study conducted its intervention at home [55], with no studies implementing community‑based deployment, highlighting a critical gap for real‑world scalability. The traditional health management model mainly relied on hospital or home-centered interventions, and the increasing demand for population aging and chronic disease management posed greater challenges to this model [63-65]. In contrast, community‑based health management was considered a sustainable strategy that could provide convenient, long‑term services and promote healthy behaviors among older adults [66,67]. Community and group‑based programs provide social interaction, emotional support, and a sense of belonging, which reduce isolation and strengthen psychological stability [16,22]. Community XR interventions also faced some challenges in resource sharing and management. Community-based XR exercise often requires multiple participants to share equipment, space, and network resources. However, most community spaces lacked the specialized infrastructure necessary to support immersive exercise programs, including adequate physical space, stable power supply, network connectivity, and safety facilities [68]. This created unresolved issues such as shared use time arrangements, equipment maintenance, and technical assistance training for community staff [68,69]. For community XR projects to be truly implemented, they also need the support of policies and management mechanisms [70]. For example, by optimizing community resource allocation and conducting technical personnel training, practical problems such as equipment sharing and insufficient infrastructure can be addressed [71].

The dominance of controlled environments in the reviewed studies, therefore, limits the interpretation of continuity and scalability outcomes. While such settings support feasibility testing and supervised delivery, they do not reflect the operational conditions under which older adults engage in exercise in everyday community contexts, making it difficult to assess whether XR-based interventions can be sustained beyond experimental or clinical settings.

### Comparison With Prior Work

The findings of this study aligned with broader existing evidence. With the application of technology-assisted interventions in the field of rehabilitation and exercise for older adults, participatory methods and user-centered design principles were gradually integrated into the entire process of intervention development [72,73]. This trend also appeared in VR rehabilitation and digital health projects, where researchers generally emphasized the widespread application of participatory design and rapid prototyping design [35]. However, our synthesis identified a critical difference: early creative ideation and comprehensive multi‑stage service design remained underused. Most studies adopted fragmented design processes without forming a closed loop between service design and adherence monitoring, which limited the ability to anticipate long‑term engagement barriers and optimize real‑world implementation.

Several studies confirmed that immersive VR interventions provided positive feedback to older adults, including heightened feelings of pleasure, increased willingness to participate in activities, and improved emotional well-being [74,75]. However, some studies reported side effects, such as fatigue, mild physical discomfort, or sensitivity to the headset after the intervention [76,77].

High short-term adherence appeared consistent with prior findings on gamified VR engagement [26,78]. However, this adherence pattern likely resulted from 2 limitations: first, the intervention duration was insufficient, and second, the adherence assessment did not form a standardized indicator, but rather participants achieved a real and sustained change in behavior. In interdisciplinary research practice, standardized compliance assessment played a crucial role. It bridged definitional gaps across disciplines, enabled unified evaluation criteria, and offered standardized measurement tools for longitudinal tracking [60,79]. Furthermore, due to the lack of follow-up data, researchers could not determine whether such adherence persisted beyond the short term [80].

However, a critical underexplored dimension in prior work and our synthesis concerned the setting of implementation. Existing research suggested that implementing XR intervention in community settings could support social interaction, emotional regulation, and behavioral improvement [81-83]. However, most studies focused on hospitals, laboratories, and care facilities. This narrow setting focus weakened the ecological validity of research findings and hindered a comprehensive evaluation of the long-term sustainability of interventions. Integrating XR into community programs could have improved accessibility, peer support, and emotional connection; however, this remained an overlooked area in current research.

### Limitations and Future Research

This review had some limitations. First, the number of studies included in the analysis was relatively small, and the sample size of most individual studies was also small. More importantly, the studies showed pronounced heterogeneity in design, baseline characteristics of participants, and intervention implementation processes. These differences not only reduced the statistical power of the overall analysis but also limited the generalizability of the research conclusions. In addition, restricting inclusion to studies with accessible full texts may have excluded relevant work based on access rather than content, introducing potential selection bias; gray literature and trial registries were not systematically searched, which may have resulted in the omission of unpublished or ongoing studies. Second, none of the included studies established longitudinal follow-up links, which made it impossible to determine whether the short-term participation adherence translated into long-term behavioral change or functional improvement. Third, regarding intervention settings, most studies focused on hospitals, care facilities, or laboratory environments. In contrast, empirical evidence from community or home settings remained relatively limited, which also weakened the applicability of the findings to real-life scenarios. Finally, although this review adopted an XR-oriented scope, all included studies used VR interventions, reflecting the current concentration of evidence rather than an intentional focus on VR, and exploration of AR or MR modes remained relatively limited. Studies applying AR or MR technologies were identified in the broader literature and have demonstrated positive physical or cognitive outcomes among older adults [84,85]; however, these approaches have not yet been systematically examined within service design-oriented frameworks. The limitations of this technology exploration made it difficult to reveal the comparative advantages of different XR methods in terms of accessibility, usability, and situational adaptability, which affected the comprehensive understanding of the overall application value of XR technology.

To address the shortcomings of current research, future studies should adopt standardized, multidimensional adherence assessment metrics [60]. By establishing unified assessment dimensions and criteria, these metrics could resolve inconsistencies caused by variations in measurement and provide a stable foundation for long-term follow-up studies. Exploration of community-based interventions should be strengthened, with designs that closely mirror real-world exercise environments to address contextual barriers to adaptation effectively. Furthermore, service design tools such as user journey maps should be incorporated into the intervention process, leveraging their visualization capabilities to identify potential dropout risk points at each stage of the intervention, providing a concrete reference for iterative optimization of intervention plans. In parallel, multimodal tracking systems should be developed that integrate physiological data collected by wearable devices, participant behavioral logs, and in-game performance metrics. These systems should generate adaptive feedback to provide participants with more personalized support for interventions. Finally, future research should expand its technological scope by exploring AR and MR modalities and evaluating their potential for more inclusive and ecologically valid exercise interventions.

### Conclusions

This review systematically evaluated how XR-based exercise interventions for older adults incorporated service design strategies to support exercise adherence. Although participatory design and gamified VR experiences have been widely used in related research, most studies have remained fragmented and short-term. There were clear deficiencies in both the systematic integration of multistage service design processes and the effective incorporation of standardized compliance frameworks; a comprehensive research practice system had not yet been established. Research findings indicated generally high levels of exercise adherence; however, conclusions varied across studies, and few focused on the intensity of adherence, long-term maintenance, or the ecological validity of the findings. Further analysis from the perspectives of intervention setting and technology application revealed that most existing interventions focused on hospital or laboratory settings, whereas the exploration of community settings, as well as the development of other XR modalities such as AR and MR, was relatively limited. To address this gap, future XR intervention research should integrate structured service design tools throughout the intervention lifecycle, develop standardized adherence metrics, establish multidimensional adherence tracking mechanisms, and extend deployment to real-life settings. By promoting synergy among behavioral engagement, service continuity, and situational adaptability, the effectiveness of XR intervention in helping older adults build sustainable exercise habits, while also providing an empirical reference for the innovative development of inclusive digital health services.

## Appendix

Multimedia Appendix 1 PRISMA checklist.

Multimedia Appendix 2 Search strategy.

Multimedia Appendix 3 Quality Assessment of the Studies Included.

## Abbreviations

AR: augmented reality
MMAT: Mixed Methods Appraisal Tool
MR: mixed reality
PRISMA: Preferred Reporting Items for Systematic Reviews and Meta-Analyses
TiSDD: This is Service Design Doing
UX: user experience
VR: virtual reality
XR: extended reality
