# Supplementary material for: Service Design Strategies to Enhance Exercise Adherence in Extended Reality Interventions for Older Adults: Systematic Review
Source: JMIR Aging. 2026 Apr 1;9:e86595. doi: 10.2196/86595 (PMC13085984; doi:10.2196/86595)
Supplement: Multimedia Appendix 2 [file aging_v9i1e86595_app2.docx]

**Multimedia Appendix 2**

The detailed search strategies for each database are provided below. Searches were conducted in July 2025, and were limited to peer-reviewed articles published in English between January 2020 and July 2025. Full texts were required for eligibility assessment and data extraction. Articles for which the full text could not be accessed were excluded at the screening stage. The searches combined keywords and subject headings (where applicable) covering XR technologies, service design methods, and older adults engaging in physical activity.

**PubMed**

("virtual reality" OR "augmented reality" OR "mixed reality" OR "extended reality" OR "immersive technology") AND ("service design" OR "user journey" OR "journey mapping" OR "experience map" OR "co-design" OR "participatory design" OR "user-centered design" OR "human-centered design") AND ("older adult" OR "elderly" OR "geriatric") AND ("exercise" OR "physical activity" OR "rehabilitation")
Filters applied: English language and publication date between January 1, 2020, and July 1, 2025.
The search yielded **7 records** in total.

**Scopus**

("virtual reality" OR "augmented reality" OR "mixed reality" OR "extended reality" OR "immersive technology") AND ("service design" OR "user journey" OR "journey mapping" OR "experience map" OR "co-design" OR "participatory design" OR "user-centered design" OR "human-centered design") AND ("older adult" OR "elderly" OR "geriatric") AND ("exercise" OR "physical activity" OR "rehabilitation") Results: [fill in number]

Filters applied: English language and publication date between January 1, 2020, and July 1, 2025.
The search yielded **924 records** in total.

**Web of Science**

("virtual reality" OR "augmented reality" OR "mixed reality" OR "extended reality" OR "immersive technology") AND ("service design" OR "user journey" OR "journey mapping" OR "experience map" OR "co-design" OR "participatory design" OR "user-centered design" OR "human-centered design") AND ("older adult" OR "elderly" OR "geriatric") AND ("exercise" OR "physical activity" OR "rehabilitation") Results: [fill in number]

Filters applied: English language and publication date between January 1, 2020, and July 1, 2025.
The search yielded **8 records** in total.

**CINAHL**

("virtual reality" OR "augmented reality" OR "mixed reality" OR "extended reality" OR "immersive technology") AND ("service design" OR "user journey" OR "journey mapping" OR "experience map" OR "co-design" OR "participatory design" OR "user-centered design" OR "human-centered design") AND ("older adult" OR "elderly" OR "geriatric") AND ("exercise" OR "physical activity" OR "rehabilitation") Results: [fill in number]

Filters applied: English language and publication date between January 1, 2020, and July 1, 2025.
The search yielded **2 records** in total.

**PsycINFO**

("virtual reality" OR "augmented reality" OR "mixed reality" OR "extended reality" OR "immersive technology") AND ("service design" OR "user journey" OR "journey mapping" OR "experience map" OR "co-design" OR "participatory design" OR "user-centered design" OR "human-centered design") AND ("older adult" OR "elderly" OR "geriatric") AND ("exercise" OR "physical activity" OR "rehabilitation") Results: [fill in number]

Filters applied: English language and publication date between January 1, 2020, and July 1, 2025.
The search yielded **6 records** in total.

**ACM Digital Library**

("virtual reality" OR "augmented reality" OR "mixed reality" OR "extended reality" OR "immersive technology") AND ("service design" OR "user journey" OR "journey mapping" OR "experience map" OR "co-design" OR "participatory design" OR "user-centered design" OR "human-centered design") AND ("older adult" OR "elderly" OR "geriatric") AND ("exercise" OR "physical activity" OR "rehabilitation") Results: [fill in number]

Filters applied: English language and publication date between January 1, 2020, and July 1, 2025.
The search yielded **26 records** in total.
