# Supplementary material for: Service Design Strategies to Enhance Exercise Adherence in Extended Reality Interventions for Older Adults: Systematic Review
Source: JMIR Aging. 2026 Apr 1;9:e86595. doi: 10.2196/86595 (PMC13085984; doi:10.2196/86595)
Supplement: Multimedia Appendix 3 [file aging_v9i1e86595_app3.docx]

| **Multimedia Appendix 3.** Critical appraisal of the methodological quality of included studies | | | | | | | |
| --- | --- | --- | --- | --- | --- | --- | --- |
| 1. Qualitative studies | | | | | | | |
| First author | Year | Citation | Q 1.1 | Q 1.2. | Q 1.3. | Q 1.4. | Q 1.5. |
| Sweeney* | 2025 |  | Yes | Yes | Yes | Yes | Yes |
| Bosch-Barceló* | 2024 |  | Yes | Yes | Yes | Yes | Yes |
| Mehrabi* | 2022 |  | Yes | Yes | Yes | Yes | Yes |
| Matsangidou* | 2022 |  | Yes | Yes | Yes | Yes | Yes |
| Matsangidou* | 2023 |  | Yes | Yes | Yes | Yes | Yes |
| Eisapour* | 2020 |  | Yes | Yes | Yes | Yes | Yes |
| Xu * | 2021 |  | Yes | Yes | Can't tell | Yes | Can't tell |
| Q 1.1. Is the qualitative approach appropriate to answer the research question? | | | | | | | |
| Q 1.2. Are the qualitative data collection methods adequate to address the research question? | | | | | | | |
| Q 1.3. Are the findings adequately derived from the data? | | | | | | | |
| Q 1.4. Is the interpretation of results sufficiently substantiated by data? | | | | | | | |
| Q 1.5. Is there coherence between qualitative data sources, collection, analysis and interpretation? | | | | | | | |

| 2. Randomised control trials | | | | | | | |
| --- | --- | --- | --- | --- | --- | --- | --- |
| First author | Year | Citation | Q 2.1. | Q 2.2. | Q 2.3. | Q 2.4. | Q 2.5. |
| Xu * | 2021 |  | Yes | Yes | Yes | Yes | Yes |
| Q 2.1. Is randomization appropriately performed? | | | | | | | |
| Q 2.2. Are the groups comparable at baseline? | | | | | | | |
| Q 2.3. Are there complete outcome data? | | | | | | | |
| Q 2.4. Are outcome assessors blinded to the intervention provided? | | | | | | | |
| Q 2.5 Did the participants adhere to the assigned intervention? | | | | | | | |
| 3. Non-randomised studies | | | | | | | |
| First author | Year | Citation | Q 3.1. | Q 3.2. | Q 3.3. | Q 3.4. | Q 3.5. |
| De Luca | 2025 |  | Yes | Yes | Yes | Can't tell | Yes |
| Sweeney* | 2025 |  | Yes | Yes | Yes | Can't tell | Yes |
| Ciemer | 2025 |  | Yes | Yes | Yes | Yes | Yes |
| Bosch-Barceló* | 2024 |  | Yes | Yes | Yes | Yes | Yes |
| Mehrabi* | 2022 |  | Yes | Yes | Yes | Yes | Yes |
| Matsangidou* | 2022 |  | Yes | Yes | Yes | Can't tell | Yes |
| Matsangidou* | 2023 |  | Yes | Yes | Yes | Can't tell | Yes |
| Eisapour* | 2020 |  | Yes | Yes | Yes | Yes | Yes |
| Q 3.1. Are the participants representative of the target population? | | | | | | | |
| Q 3.2. Are measurements appropriate regarding both the outcome and intervention (or exposure)? | | | | | | | |
| Q 3.3. Are there complete outcome data? | | | | | | | |
| Q 3.4. Are the confounders accounted for in the design and analysis? | | | | | | | |
| Q 3.5. During the study period, is the intervention administered (or exposure occurred) as intended? | | | | | | | |

| 5. Mixed methods studies | | | | | | | |
| --- | --- | --- | --- | --- | --- | --- | --- |
| First author | Year | Citation | Q 5.1. | Q 5.2. | Q 5.3. | Q 5.4. | Q 5.5. |
| Sweeney* | 2025 |  | Yes | Yes | Yes | Yes | Yes |
| Bosch-Barceló* | 2024 |  | Yes | Yes | Yes | Yes | Yes |
| Mehrabi* | 2022 |  | Yes | Yes | Yes | Yes | Can't tell |
| Matsangidou* | 2022 |  | Yes | Yes | Yes | Yes | Yes |
| Matsangidou* | 2023 |  | Yes | Yes | Yes | Yes | Can't tell |
| Eisapour* | 2020 |  | Yes | Yes | Yes | Yes | Yes |
| Xu * | 2021 |  | Yes | Yes | Yes | Yes | Can't tell |
| Q 5.1. Is there an adequate rationale for using a mixed methods design to address the research question? | | | | | | | |
| Q 5.2. Are the different components of the study effectively integrated to answer the research question? | | | | | | | |
| Q 5.3. Are the outputs of the integration of qualitative and quantitative components adequately  interpreted? | | | | | | | |
| Q 5.4. Are divergences and inconsistencies between quantitative and qualitative results adequately addressed? | | | | | | | |
| Q 5.5. Do the different components of the study adhere to the quality criteria of each tradition of the  methods involved? | | | | | | | |

*****mixed methods studies. Following the instruction of the MMAT guidance the mixed-methods studies first were assessed on their qualitative and quantitative components independently, and finally using the questions

- 1. – 5.5. on their mixed-methods methodology.

**Note: all studies answered ‘yes’ to the first two screening questions of the MMAT:**

- 1. Are there clear research questions?
  2. Do the collected data allow to address the research questions?

**reference**

1. Matsangidou M, Frangoudes F, Hadjiaros M, Schiza E, Neokleous KC, et al. “Bring me sunshine, bring me (physical) strength”: The case of dementia. Designing and implementing a virtual reality system for physical training during the covid-19 pandemic. Int J Hum-Comput Stud. 2022;165:102840. doi: 10.1016/j.ijhcs.2022.102840.

2. Matsangidou M, Frangoudes F, Schiza E, Neokleous KC, Papayianni E, et al. Participatory design and evaluation of virtual reality physical rehabilitation for people living with dementia. Virtual Real. 2023;27(1):421-438. doi: 10.1007/s10055-022-00639-1.

3. Sweeney G, Boyd F, Keogh M, Lyczba P, Forrest E, et al. A technology-enriched approach to increasing rehabilitation dose after stroke: Clinical feasibility study. Clin Rehabil. 2025. doi: 10.1177/02692155251333542.

4. Bosch-Barceló P, Masbernat-Almenara M, Martínez-Navarro O, Tersa-Miralles C, Pakarinen A, et al. A gamified virtual environment intervention for gait rehabilitation in parkinson’s disease: Co-creation and feasibility study. J NeuroEng Rehabil. 2024;21(1):107. doi: 10.1186/s12984-024-01399-6.

5. Mehrabi S, Muñoz JE, Basharat A, Boger J, Cao S, et al. Immersive virtual reality exergames to promote the well-being of community-dwelling older adults: Protocol for a mixed methods pilot study. JMIR RES Protoc. 2022;11(6):e32955. doi: 10.2196/32955.

6. Eisapour M, Cao S, Boger J. Participatory design and evaluation of virtual reality games to promote engagement in physical activity for people living with dementia. J Rehabil Assist Technol Eng. 2020;7:2055668320913770. doi: 10.1177/2055668320913770.

7. Xu Y, Tong M, Ming W-K, Lin Y, Mai W, et al. A depth camera–based, task-specific virtual reality rehabilitation game for patients with stroke: Pilot usability study. JMIR Serious Games. 2021;9(1):e20916. doi: 10.2196/20916.

8. Ciemer C, Schott N, Klotzbier TJ, Ghellal S. Effectiveness, enjoyment, and meaningfulness of a virtual reality gait-based fall prevention exergame in community-dwelling healthy older adults: An interdisciplinary pilot study. Front Psychol. 2025;16. doi: 10.3389/fpsyg.2025.1610377.

9. De Luca V, Qbilat M, Cuomo A, Bianco A, Cesaroni F, et al. Virtual reality solution to promote adapted physical activity in older adults: Outcomes from vr2care project exploratory study. Front Public Health. 2025;13. doi: 10.3389/fpubh.2025.1584406.
